# Bio-strengthening of cementitious composites from incinerated sugarcane filter cake by a calcifying bacterium *Lysinibacillus* sp. WH

**DOI:** 10.1038/s41598-022-11330-5

**Published:** 2022-04-29

**Authors:** Zerlinda Mara Ditta, Nantawat Tanapongpisit, Wittawat Saenrang, Ittipon Fongkaew, Poemwai Chainakun, Wasan Seemakram, Sophon Boonlue, Vanchai Sata, Jindarat Ekprasert

**Affiliations:** 1 Biological Science Program, Faculty of Science, Khon Kaen University, Khon Kaen, 40002 Thailand; 2 School of Physics, Institute of Science, Suranaree University of Technology, Nakhon Ratchasima, 30000 Thailand; 3 Center of Excellence in Advanced Functional Materials, School of Physics, Suranaree University of Technology, Nakhon Ratchasima, 30000 Thailand; 4 Department of Microbiology, Faculty of Science, Khon Kaen University, 123 Mitraparp Rd, Muang, Khon Kaen, 40002 Thailand; 5 Protein and Proteomics Center for Commercial and Industrial Purposes, Faculty of Science, Khon Kaen University, Khon Kaen, 40002 Thailand; 6 Sustainable Infrastructure Research and Development Center, Faculty of Engineering, Khon Kaen University, Khon Kaen, 40002 Thailand

**Keywords:** Biomaterials, Environmental biotechnology, Industrial microbiology, Biotechnology, Materials science, Structural materials

## Abstract

This study investigated Microbially Induced Calcite Precipitation (MICP) technology to improve the mechanical properties of cementitious composites containing incinerated sugarcane filter cake (IFC) using a calcifying bacterium *Lysinibacillus* sp. WH. Both IFC obtained after the first and second clarification processes, referred to as white (IWFC) and black (IBFC), were experimented. This is the first work to investigate the use of IBFC as a cement replacement. According to the X-ray fluorescence (XRF) results, the main element of IWFC and IBFC was CaO (91.52%) and SiO_2_ (58.80%), respectively. This is also the first work to investigate the use of IBFC as a cement replacement. We found that the addition of strain WH could further enhance the strength of both cementitious composites up to ~ 31%, while reduced water absorption and void. Microstructures of the composites were visualized using a scanning electron microscope (SEM). The cement hydration products were determined using X-ray diffraction (XRD) followed by Rietveld analysis. The results indicated that biogenic CaCO_3_ was the main composition in enhancing strength of the IBFC composite, whereas induce tricalcium silicate (C_3_S) formation promoting the strength of IWFC composite. This work provided strong evidence that the mechanical properties of the cementitious composites could be significantly improved through the application of MICP. In fact, the strength of IFC-based cementitious composites after boosting by strain WH is only 10% smaller than that of the conventional Portland cement. While using IFC as a cement substitute is a greener way to produce environmentally friendly materials, it also provides a solution to long-term agro-industrial waste pollution problems.

## Introduction

Cement has become one of the most widely used building materials in the last century due to its durability and low cost^1^. However, a rise in cement consumption has resulted in a global environmental problem. Cement production accounts for approximately 6% of total anthropogenic CO_2_ emissions and 50% of total CO_2_ emissions from the building construction and operation sector worldwide. The high amount of CO_2_ in the atmosphere is due to an intensive use of energy during kiln production process (70–80%) and a consumption of non-renewable resources, causing global warming^2–4^.

With growing concern about reducing the environmental problem caused by CO_2_ emissions, as well as prolonging life and improving durability of cement-based materials, biocement has emerged as a potential solution. Several agro-industrial wastes can be used as cement substitutes such as sugarcane straw ash^5^, rice husk ash^6^, wheat straw ash^7^, corn cob ash^8^, wood shaving^9^, paper mill sludge ash, sugarcane bagasse ash^10^ and sugarcane filter cake from juice clarification processes in the sugar industry^11^.

After rice and rubber trees, the sugar industry is crucial to Thailand's economic development^12^. Thailand is one of the top five sugar producing countries in the world^13^. There are 16 sugar factories in the Northeast, and the sugar industry is the region's major focus^12^. During sugar production, one of the main solid wastes is sugarcane filter cake^14,15^. In this study, we would like to use sugarcane filter cake as a cement replacement because the sugar industry generates a large amount of filter cakes as waste. There are two different types of filter cakes inspected here, including the one obtained from the first and the second clarification processes, which are referred to, based on their color, as “black” and “white” filter cake, respectively. While white filter cake has been used as a cement clinker and cement replacement in concrete^16,17^, the study on engineering applications of the black filter cake is still limited (e.g. no applications for producing value-added products).

Sua-Iam and Makul^11^ investigated the use of Incinerated Sugarcane Filter Cake (IFC) by looking at its effects on the properties of self-compacting concrete mixed with ordinary Portland cement as a cement replacement. They used IFC at 10, 20, 30 and 40% by weight of cement. Their results showed that when IFC was added to the cement mixture, the density and compressive strength of the self-compacting concrete (SCC) mixture decreased noticeably in all conditions. Furthermore, when cement was replaced with IFC, the water absorption of the hardened SCC after curing treatment was significantly greater than that of the control concrete (without IFC replacement). Although using IFC as a cement replacement is, of course, more environmentally friendly, it is clear that the qualities of the materials become lower than those without IFC replacement.

Much recent research has focused on the use of biological substances to improve the mechanical properties of cementitious materials, particularly calcifying bacteria, or what is known as microbially induced calcium carbonate precipitation (MICP)^18–21^. A diverse range of bacterial groups play an important role in MICP and have the potential to be used as biocement additives and self-healing agents in cementitious materials. Microbial repair mechanisms occur through the bio-deposition of CaCO_3_, demonstrating an improvement in the mechanical and durability properties of cement-based materials. Moreover, MICP bacteria can withstand mechanical stress during cement mixing and remain viable in high alkalinity environments, which is a characteristic of cement materials^22^. Furthermore, the use of this biological repair technology is highly preferred because the mineral precipitation generated by bacterial activities is safer to the environment than its chemical counterpart^22,23^. Due to its environmentally friendly nature, we are interested in using calcifying bacteria to enhance the strength of the cement partially replaced by IFC. In addition, many topics have been discussed about MICP bacteria mostly for concrete and mortar application, but very limited studies have conducted in the cementitious composite systems, especially when agro-industrial wastes are incorporated.

One of the most important characteristics of *Lysinibacillus* spp. is that they have the ability to form spores that can survive under harsh environments (i.e. high alkalinity, limited nutrients, etc.) such as in the building materials. Furthermore, despite the fact that urea hydrolysis is the simplest of these metabolic processes and its pathway has received the most attention due to its efficiency for MICP productivity, urea hydrolysis generates ammonia as a byproduct, raising the risk of steel corrosion in reinforced concrete. As a result, non-ureolytic reactions become more appealing for practical applications*.* There have only been a few reports of bacterial genus *Lysinibacillus* spp. being capable of precipitating CaCO_3_^19,24–28^. Ekprasert et al.^19^ studied the mechanical properties of biocement containing a newly isolated *Lysinibacillus* sp. WH capable of precipitating CaCO_3_ via non-ureolytic processes by using calcium acetate, calcium chloride and calcium nitrate as a calcium source. Note that strain WH was grown in the absence of urea, the possible mechanism for it to precipitate CaCO_3_ is likely to be the deamination of yeast extract presented in B4 medium^29^. They found that *Lysinibacillus* sp. WH could increase the compressive strength of cement by 40–50% compared to the control (Portland cement). Therefore, due to the ability of strain WH to enhance compressive strength of the cement, it is assumed that the incorporation of this bacterium into the cement composites in this work would help compensate the loss of strength as a result of IFC replacement.

Moreover, to the best of our knowledge, previous literature revealed no studies on the production of biocement incorporating both IFC as a cement replacement and calcifying bacteria from the genus *Lysinibacillus* spp. as a cement addition. The aim of this study is, therefore, to investigate the effects of biogenic CaCO_3_ produced by *Lysinibacillus* sp. WH on the physical properties, including compressive strength, water absorption and void of biocement containing IFC (both white and black filter cake). We then discussed the results towards the potential use of filter cake as a cement replacement in biocement application.

## Methods

### Preparation of incinerated sugarcane filter cake

Black and white sugarcane filter cakes were obtained from Khon Kaen Sugar Industry Public Company Limited in Khon Kaen and Erawon Sugar Company Limited in Nong Bua Lamphu, Thailand, respectively. Both types of filter cakes were incinerated in an electrical furnace at 850 ± 20 °C for 3 h by increasing the temperature at a rate of 10 °C/min. The incinerated black and white sugarcane filter cake (IFC) were allowed to cool at room temperature before sieving using a sieve with a mesh size of ≤ 5 mm.

### Preparation of bacterial culture for mixing into biocement

*Lysinibacillus* sp. strain WH used in this study was isolated from saline soil samples collected from an abandoned paddy field in Surin province, Thailand. Genus identification of this bacterium was carried out based on its 16S rRNA sequence as previously reported by Ekprasert et al.^19^. *Lysinibacillus* sp. strain WH was grown in B4 medium (per liter: 4 g yeast extract, 5 g dextrose, 2.5 g calcium acetate, adjust pH to 8.2)^30^ to induce CaCO_3_ precipitation. The pH of the B4 medium was adjusted from 7 to 8.2 by adding 1 N NaOH in order to obtain an optimum pH for CaCO_3_ precipitation. The cultures were incubated with shaking at 150 rpm, 30 °C for 4 days, which was the optimum incubation time for CaCO_3_ precipitation^19^. Then, the cultures were centrifuged at 8000 rpm for 15 min to collect cell pellets. Concentrations of bacterial cultures were quantified using a plate count method prior to use in biocement.

### Biocement preparation

Biocement samples were set up into 6 treatments as indicated in Table 1. In the treatment which Ordinary Portland cement (OPC) was replaced, the replacement ratio was 10% w/w of either incinerated white filter cake (IWFC) or incinerated black filter cake (IBFC). Bacterial culture concentration was determined by the plate count method. Then, 1 mL of 10^8^ CFU of bacteria, which were mostly vegetative cells, was added into each cement cube mixture. Biocement in each treatment was set up in triplicate per sampling time per analysis. OPC, bacterial cells and IFC were mixed with tap water at a water:cement ratio of 0.5. The cement mixture was casted in a cement mold size of 50 × 50 × 50 mm^3^ and then allowed to harden at room temperature for 24 h. The sample cubes were demolded and cured in tap water for 28 days. Biocement cubes were sampled at the age of 1, 7, 14 and 28 days for water absorption and compressive strength tests.

**Table 1:** Compositions of cementitious composites in each treatment.

Treatment	Composition
1	OPC
2	OPC + WH
3	OPC + IWFC
4	OPC + IWFC + WH
5	OPC + IBFC
6	OPC + IBFC + WH

### Water absorption test and determination of volume of permeable pore space (voids)

Water absorption test was conducted according to the ASTM C642-13 standard procedure^31^. Briefly, tree replicate of biocement specimens were dried in a hot-air oven at 110 ± 5 °C for 24 h. The specimens were cooled down and dried at a temperature of 20 to 25 °C prior to weighing (*The Oven-Dry-Mass Value (A))*. After that, the specimens were immersed in tap water for 48 h at a temperature of 21 °C. The surface of the specimens was dried using a water absorbent towel prior to determining *the Saturated Mass After Immersion (B)*. Then, the specimens were placed in a container, covered with tap water, and boiled for 5 h. The specimens were cooled down for at least 14 h or until their temperature was approximately 20–25 °C. The surface moisture was removed with a towel and the mass of the specimens was determined as the *Saturated Mass After Boiling (C).* After immersion and boiling in the tap water, the specimens were weighed under water to determine the *Immersed Apparent Mass value (D).* The water absorption and void were calculated using the Eqs. (1) and (2) as follows:

1 $$Water absorption \left( \% \right) = \left[ {\frac{{\left( {B - A} \right)}}{A}} \right] \times 100$$

2 $$Voids \left( \% \right) = \frac{{\left( {C - A} \right)}}{{\left( {C - D} \right)}} \times 100$$

### Compressive strength test

Cement cube samples (three replicate) were tested for compressive strength according to ASTM C109 standard^32^ using a CBN compression testing machine (CBN Testing Corporation, Thailand). The crushed cements were powdered for Scanning Electron Microscope (SEM) and X-Ray Diffraction (XRD) analysis.

### Scanning electron microscope (SEM)

Precipitates from 4-day bacterial culture were collected by centrifugation. Then, biogenic calcite was separated from bacterial cells by filtration through a Whatman No.1 filter paper (Whatman, Merck, Germany). The precipitates left on the filter were washed with sterile water and then dried in a hot air oven at 45 °C until completely dry before being examined with a scanning electron microscope (SEM). SEM analysis was performed with OPC, biogenic CaCO_3_, IWFC, IBFC and the biocement specimens at the age of 28 days. Field Emission Scanning Electron Microscopy (FESEM; FEI Model, Helios NanoLab G3 CX, USA) was used to visualize crystal morphology of the samples.

### Quantitative XRD analysis

X-ray diffraction (XRD) analysis was used to determine the mineralogical compositions of the cement hydration products in biocement samples. The cement specimens were grounded to powder and then subjected to XRD analysis using X-ray diffractometer (Bruker D2 Phaser, USA). The spectra were scanned in a range of 10°–80°. Then, the ratio of each phase was determined according to the Rietveld refinement method using Profex software.

### Physical and chemical analysis

The elemental compositions of ordinary Portland cement (OPC), fresh black sugarcane filter cake (BFC), fresh white sugarcane filter cake (WFC), black incinerated sugarcane filter cake (IBFC) and white incinerated sugarcane filter cake (IWFC) were determined using X-Ray Fluorescence (XRF) (Horiba XGT-5200 X-Ray Analytical Microscope, UK). Physical characteristics including loss on ignition (LOI) and surface area and pore size distribution of those materials were analyzed using Thermogravimetric (TGA) and Brunauer–Emmett–Teller (BET) analysis. TGA was performed using a Thermogravimetric analyzer (Mettler Toledo Model TGA/DSC1, USA) under nitrogen gas with heating from 25 to 600 °C at a rate of 10 °C/min^33^. BET analysis was carried out using a BET analyzer (Bell Model Belsorp mini, Japan) using N_2_ gas as gaseous adsorbate.

### Statistical analysis

Analysis of Variance (ANOVA) based on the least significant difference (LSD) at *p*-value of 0.05 was used to determine significant differences among the mean values. The Statistix 10.0 program was used for all statistical analysis.

## Results

### Physical and chemical properties of raw materials

Chemical compounds in our raw materials based on the results of an X-Ray fluorescence (XRF) analysis are presented in Table 2. The main compositions of OPC were CaO (62.49%) and SiO_2_ (25.22%) with minor ratios of other compounds. XRF analysis results also showed that IWFC consisted mainly of CaO (91.52%), which is also the main component of cement. Therefore, it is reasonable to be used as a cement replacement. Differently, IBFC mainly consisted of SiO_2_ (58.80%) and a small amount of CaO (7.69%) with a minority of other constituents. The results obtained from thermogravimetric analysis indicated that the loss on ignition (LOI) of OPC, IWFC and IBFC were 4.91%, 8.56% and 0.30%, respectively. Moreover, Brunauer–Emmett–Teller (BET) analysis showed that both IWFC and IBFC had smaller specific surface areas (0.97 cm^2^/g and 0.52 cm^2^/g, respectively) than the OPC (1.06 cm^2^/g). However, mean pore diameter and total pore volume of IWFC and IBFC were less than those of the OPC. In this regard, IWFC had a smaller pore diameter (29.60 nm) but a larger total pore volume (7.1610^–3^ cm^3^/g) than those of the IBFC (39.29 nm and 5.1110^–3^ cm^3^/g). These results suggested that IWFC was more porous than IBFC.

**Table 2:** Chemical compositions and physical properties of the OPC (ordinary Portland cement); WFC (White filter cake); IWFC (Incinerated white filter cake); BFC (Black filter cake) and IBFC (Incinerated black filter cake).

	OPC	WFC	IWFC	BFC	IBFC
**Chemical analysis (%) (Mean ± standard deviation)**					
SiO_2_	25.22 ± 0.30	ND	1.91 ± 0.02	65.48 ± 0.44	58.80 ± 2.99
SO_3_	4.52 ± 0.20	1.33 ± 0.02	2.28 ± 0.01	1.40 ± 0.11	0.33 ± 0.06
K_2_O	0.56 ± 0.01	ND	ND	1.44 ± 0.10	1.20 ± 0.03
CaO	62.49 ± 0.25	89.46 ± 0.94	91.52 ± 0.12	5.07 ± 0.28	7.69 ± 0.76
TiO_2_	0.36 ± 0.01	ND	0.03 ± 0.01	1.33 ± 0.12	0.59 ± 0.08
MnO_2_	0.09 ± 0.01	ND	ND	0.62 ± 0.01	0.75 ± 0.04
Fe_2_O_3_	6.56 ± 0.14	0.41 ± 0.07	0.46 ± 0.03	7.64 ± 0.55	2.95 ± 0.17
CuO	0.08 ± 0.00	ND	0.02 ± 0.00	ND	ND
ZnO	0.08 ± 0.00	ND	ND	ND	ND
SrO	0.05 ± 0.00	ND	0.02 ± 0.00	ND	ND
Na_2_O	ND	2.33 ± 0.46	ND	ND	4.66 ± 0.41
MgO	ND	1.10 ± 0.21	1.94 ± 0.17	ND	3.56 ± 0.59
Al_2_O_3_	ND	0.45 ± 0.05	0.63 ± 0.03	11.50 ± 0.26	6.52 ± 0.49
PdO	ND	4.05 ± 0.30	ND	ND	ND
YbO	ND	0.88 ± 0.03	1.21 ± 0.02	ND	ND
P_2_O_5_	ND	ND	ND	5.47 ± 0.58	12.84 ± 1.41
Cl	ND	ND	ND	0.05 ± 0.02	ND
**Physical properties**					
LOI (%)	4.91	21.22	8.56	43.04	0.30
BET surface area (cm^2^/g)	1.06	8.19	0.97	1.49	0.52
Mean pore diameter (nm)	48.00	31.51	29.60	60.68	39.29
Total pore volume (cm^3^/g)	1.28 $\times$ 10^–2^	6.45 $\times$ 10^–2^	7.16 $\times$ 10^–3^	2.26 $\times$ 10^–2^	5.11 $\times$ 10^–3^

Chemical compounds that are not detected are denoted as ND (not detected).

**Figure 1:**
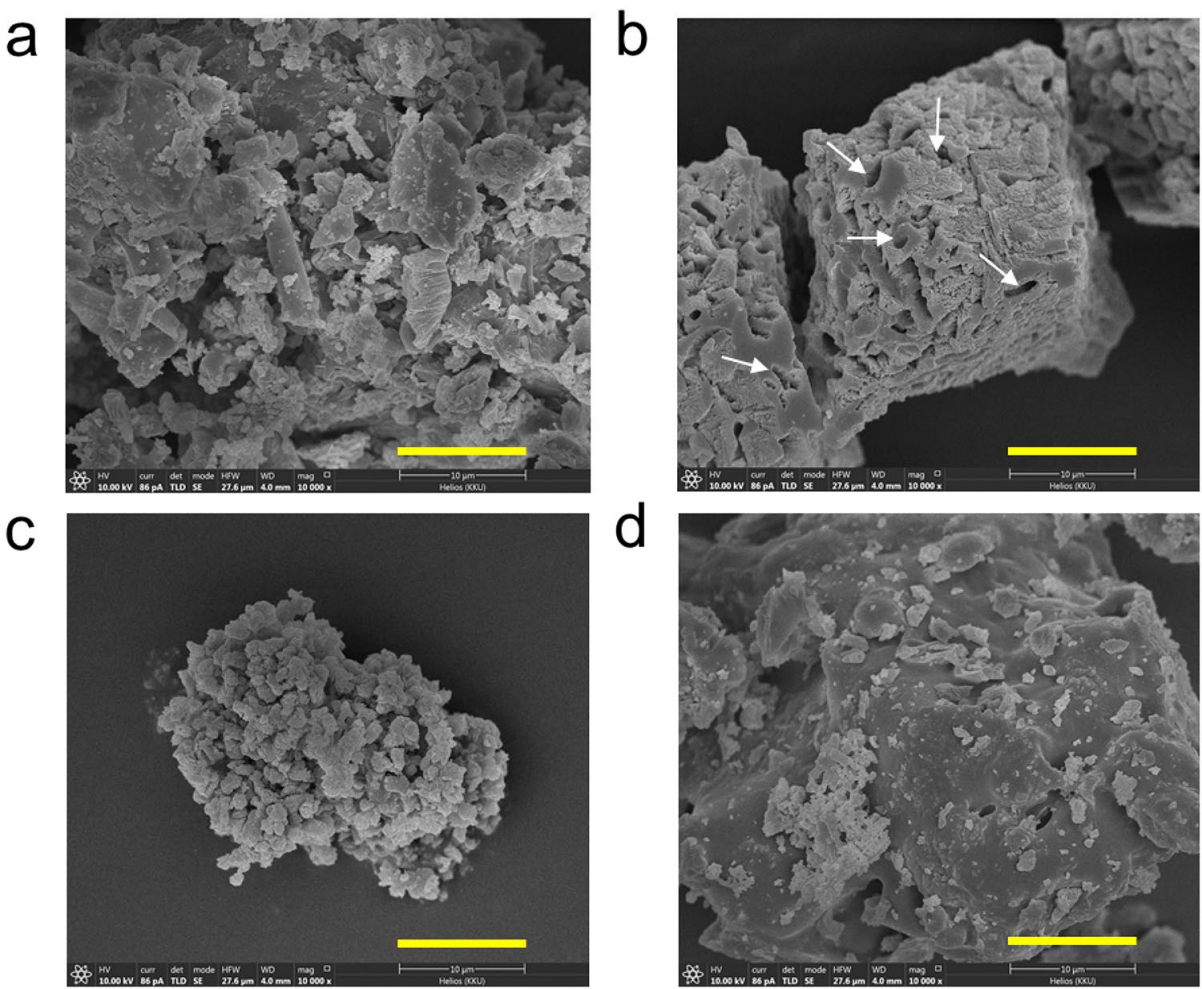
SEM micrographs (10,000X magnification) of raw materials used for producing cementitious composites; (**a**): OPC powder, (**b**): biogenic CaCO_3_ produced by *Lysinibacillus* sp. strain WH, (**c**): IWFC powder and (**d**): IBFC powder. White arrows indicate bacterial cell imprints.

Surface morphology of OPC, IWFC, IBFC and also biogenic CaCO_3_ is shown in Fig. 1. The size of all raw materials was approximately in the same range of ~ 10–50 µm with a rough surface. Biogenic CaCO_3_ crystals produced by strain WH (Fig. 1b) were in the form of calcite with porous surface due to bacterial cell imprints, which agreed with our previous study^19^.

### Compressive strength test result

**Figure 2:**
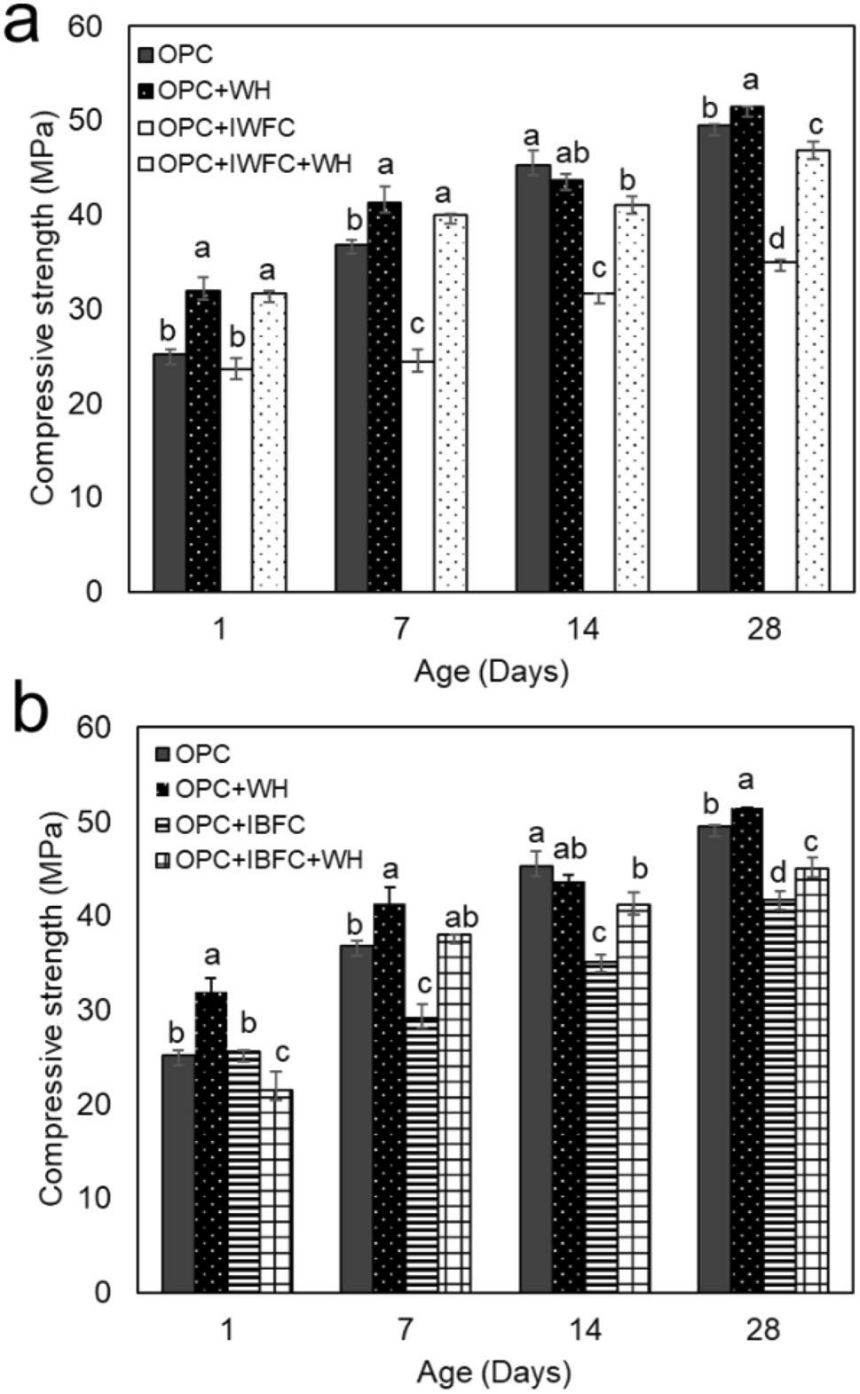
Compressive strength of cementitious composites containing incinerated white filter cake (IWFC) (**a**) and incinerated black filter cake (IBFC) (**b**). Error bars represent standard deviations of the data. Different letters above the bars indicate significant difference at *p*-value < 0.05 when compared using the Least Significant Difference method (LSD). Data with different letters are significantly different.

Compressive strength of cementitious composites was determined at the age of 1, 7, 14 and 28 days, as in e.g. Pavlík et al.^34^, Chindaprasirt et al.^35^, Mawardi et al.^36^. The results showed that the cement strength increased with increasing curing time in all specimens (Fig. 2). After 28 days of curing, the highest strength of cement was found in the OPC+WH (51.42 MPa), ensuring the ability of strain WH and its biogenic CaCO_3_ to enhance strength of the cement. It was found that the incorporation of both types of IFC (OPC+IWFC and OPC+IBFC) in cement resulted in a significant decrease in compressive strength at the age of 28 days when compared to the strength of the OPC. As expected, the replacement of IFC negatively affected the compressive strength of Portland cement. Interestingly, a reduction of cement strength due to IFC replacement could be overcome by the addition of strain WH and its CaCO_3_. It was found that OPC+IWFC+WH had a compressive strength of ~ 31% greater than that of the OPC+IWFC (Fig. 2a). Likewise, the strength of the OPC+IBFC+WH was ~ 12% higher than that of OPC+IBFC at 28 days of age (Fig. 2b). Moreover, the addition of strain WH could significantly increase compressive strength of the OPC+IWFC and the OPC+IBFC at the age of 7 days. This suggested that biogenic CaCO_3_ from strain WH is responsible for early strength of cement.

### Water absorption test result

**Figure 3:**
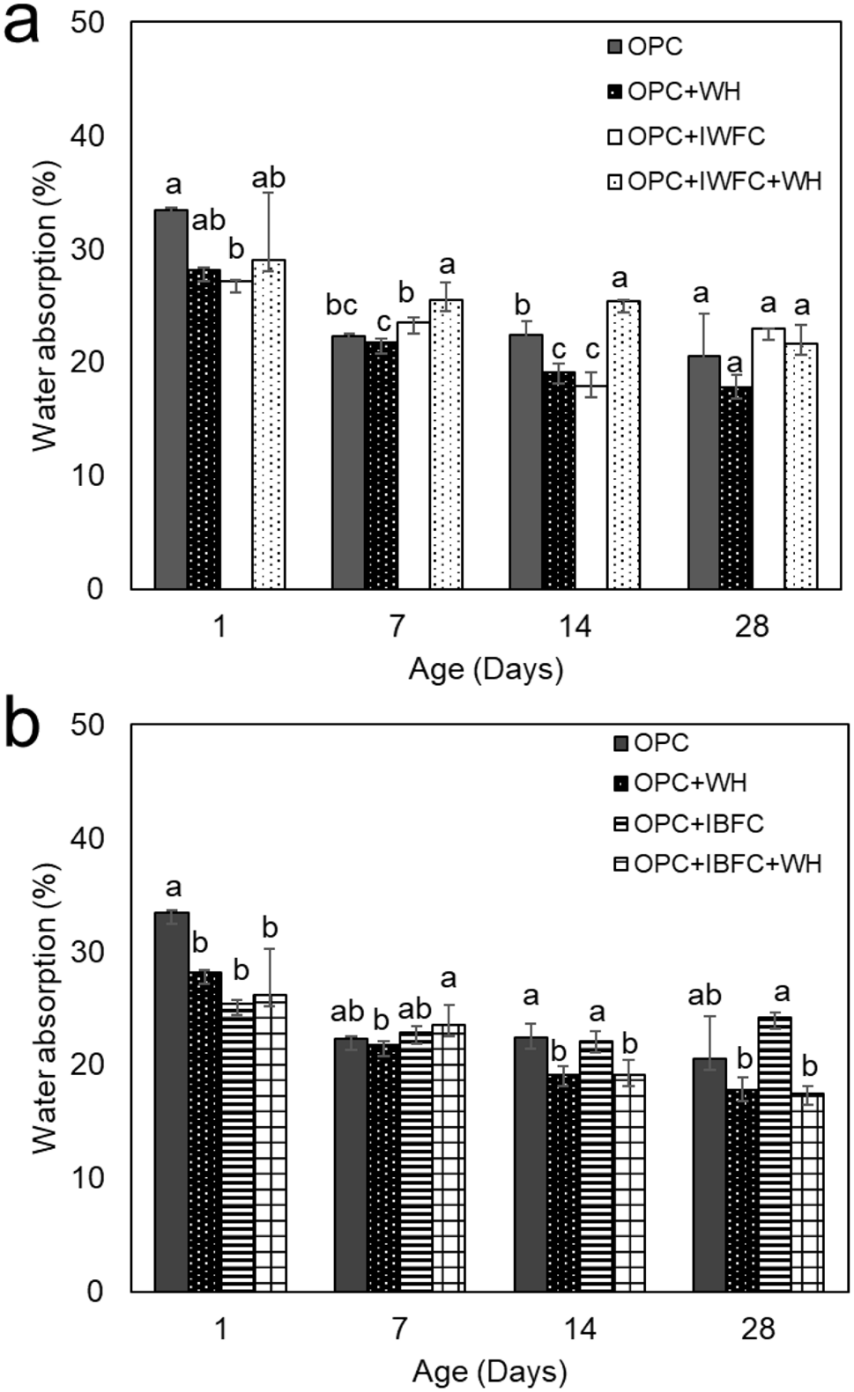
Water absorption of cementitious composites containing IWFC (**a**) and IBFC (**b**). Error bars represent standard deviations of the data. Different letters above the bars indicate significant difference at *p*-value < 0.05 when compared using the Least Significant Difference method (LSD). Data with different letters are significantly different.

Figure 3 showed the effects of strain WH on the water absorption of cementitious composites at different curing times. The water absorption of all specimens decreased with increasing curing time. The level of water absorption of the OPC+IWFC and the OPC+IBFC was not significantly different from that of the OPC. This suggested that the partial replacement of cement with either IWFC or IBFC (10% by weight) did not affect water absorption properties of the cement. It was found that the presence of strain WH in cement (OPC+WH) caused a reduction in water absorption to ~ 18%, which accounted for ~ 15% reduction compared to the control (OPC). Similarly, the effect of strain WH to reduce water absorption was also pronounced with the cement partially replaced with IFC. The results showed that, after 28 days of curing, the OPC+IWFC+WH had a water absorption of ~ 6% less than that of the OPC+IWFC (Fig. 3a). Interestingly, the addition of strain WH into the OPC+IBFC (OPC+IBFC+WH) caused ~ 28% reduction in water absorption when compared to that of the OPC+IBFC at the age of 28 days (Fig. 3b). Accordingly, the water absorption of the OPC+IWFC+WH and the OPC+IBFC + WH was even lower than that of the conventional cement (OPC). These results strongly suggest that strain WH played an important role in reducing water absorption of either cement or cement replaced with IFC.

### Volume of permeable pore space (Void) result

**Figure 4:**
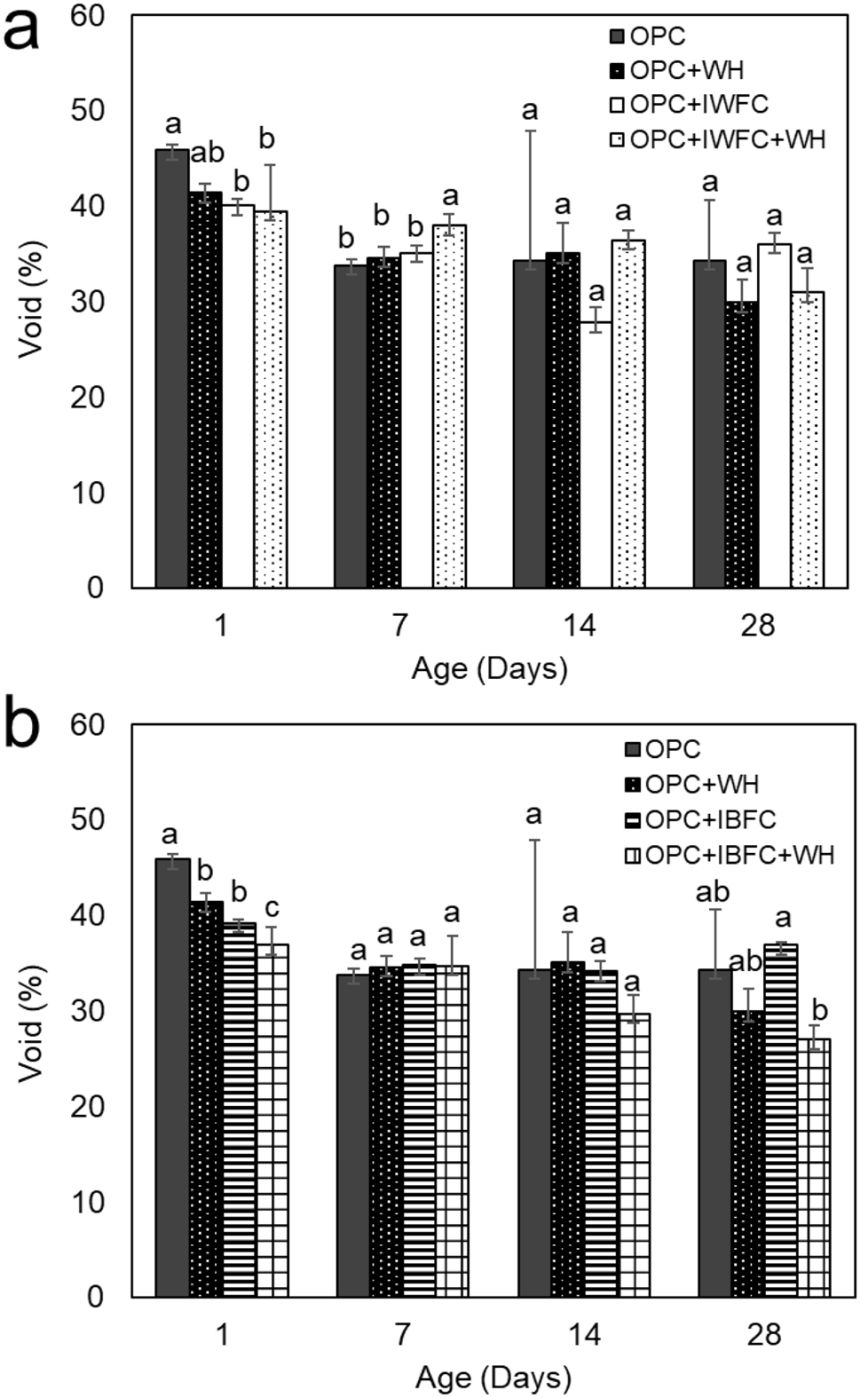
Void (%) of cementitious composites containing IWFC (**a**) and IBFC (**b**). Error bars represent standard deviations of the data. Different letters above the bars indicate significant difference at *p*-value < 0.05 when compared using the Least Significant Difference method (LSD). Data with different letters are significantly different.

The minimum void of cementitious material is a desirable property reflecting the durability of the material. The %void of all biocements is presented in Fig. 4. The results showed that %void decreased with curing time increased. The void ratio of OPC+WH (~ 30%) was significantly lower than that of the OPC (~ 34%) at 28-day age, suggesting that strain WH had a potential to reduce void in the cement. The replacement of cement by either IWFC or IBFC (OPC+IWFC and OPC+IBFC) caused an increase in %void when compared to the OPC even after 28 days of curing. This was probably because the shapes of both IFC particles (see SEM images in Fig. 1c and d) were irregular, resulting in spaces in between microparticles due to their incomplete compaction. Nevertheless, we found that the addition of strain WH into the materials could help reduce those voids. In this regard, the OPC+IWFC+WH exhibited significantly less void than the OPC+IWFC (Fig. 4a), which accounted for ~ 14% reduction in void ratio. Likewise, a notably decrease of ~ 27% in void ratio was found in the OPC+IBFC+WH, when compared to the OPC+IBFC (Fig. 4b) at the age of 28 days. The %void of ~ 31% and ~ 27% of the OPC+IWFC+WH and OPC+IBFC+WH, respectively, were even lower than that of the OPC (34%). This suggested that the added strain WH could efficiently fill pore space within the cement either with or without IFC replacement. All of these results indicated that although the replacement of cement with IFC might demote the physical properties of hardened cement, the addition of strain WH could effectively enhance those properties to its original status (i.e., water absorption and void), and even better than the conventional cement (OPC).

### Scanning electron microscope analysis of cementitious composites

**Figure 5:**
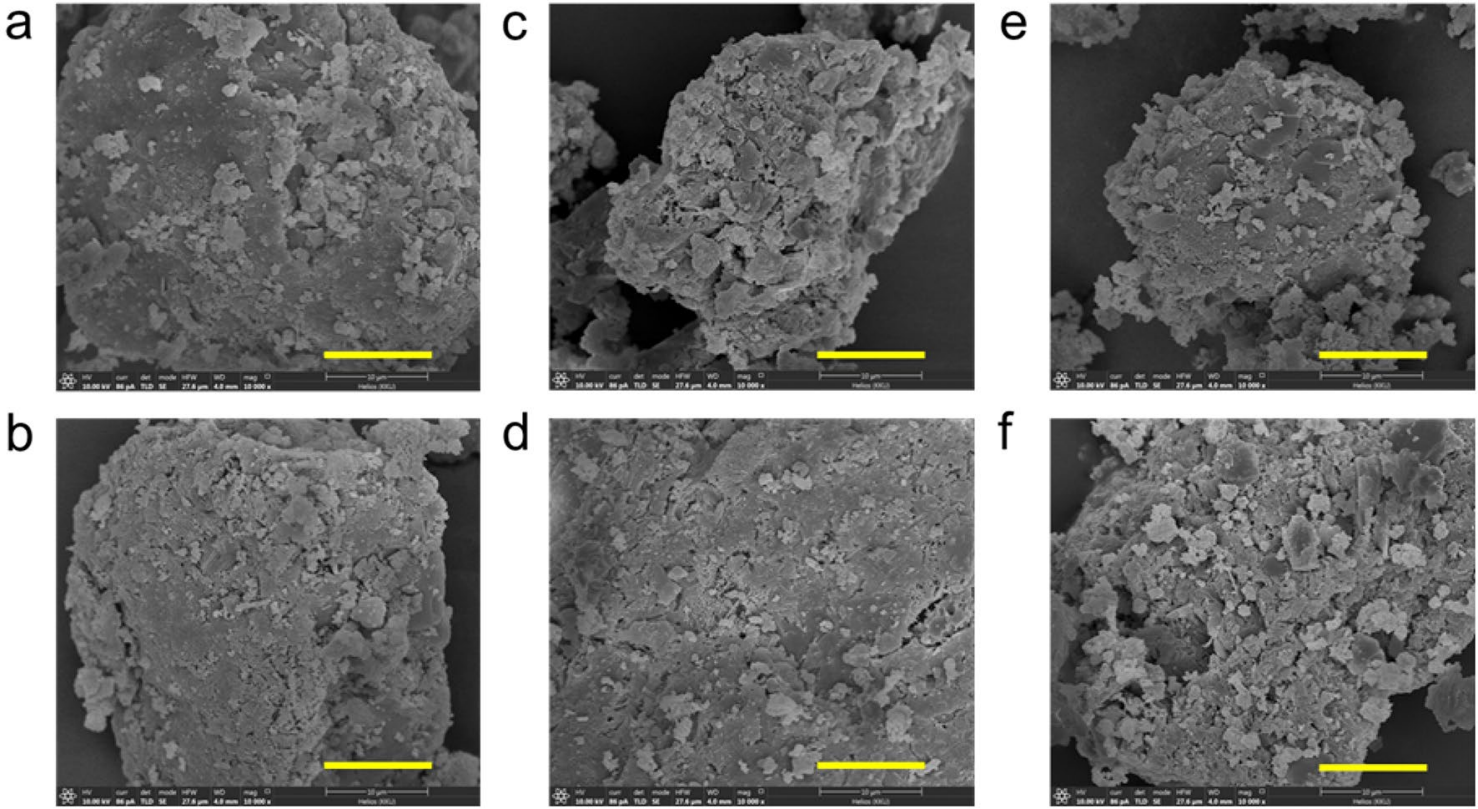
SEM micrographs (10,000X magnification) of cementitious composites at the age of 28 days; (**a**): OPC, (**b**): OPC+WH, (**c**): OPC+IWFC, (**d**): OPC+IWFC+WH, (**e**): OPC+IBFC and (**f**): OPC+IBFC+WH.

Figure 5 illustrated SEM images of the hardened cement of all treatments. It was evidenced that the cement added with strain WH (Fig. 5b, d and f) had relatively larger particle sizes than their corresponding cement without bacterial addition. This could explain how biogenic calcium carbonate affects cement strength. Biogenic CaCO_3_ might interact with alite causing an acceleration of C_3_S formation, as evidenced by an increase in the size of the microaggregates constituting the biocement. Furthermore, as a cement additive, biogenic calcium carbonate could have a filler effect, in which pores within the cement materials were plugged. As a result, the incorporation of bacterial cells and their biogenic CaCO_3_ in biocement could explain the increase in biocement strength. To investigate how strain WH affected cement hydration products, quantitative XRD analysis was performed, which is presented in the next section.

### Quantitative of X-ray diffraction analysis (XRD) of cementitious composites.

The Rietveld refinement analysis was performed to determine ratios of each cement hydration product in cementitious composites. Table 3 showed that biogenic CaCO_3_ in the OPC+WH specimen could induce the formation of ettringite via the hydration of tricalcium aluminate (C_3_A), as evidenced by a decrease in gypsum and an increase in ettringite when compared to those of the OPC. The use of IWFC, which is mostly composed of CaO, as a cement replacement caused an increase in the ettringite content of the OPC+IWFC when compared to that of the OPC. The presence of biogenic CaCO_3_ from strain WH in the OPC+IWFC+WH likely reduced carbonation of portlandite into CaCO_3_, while induced the formation of C_3_S (alite). In the case of cementitious composite containing IBFC, the results showed that the material had a lower amount of calcite but a slightly higher amount of C_3_S than those of the OPC. An excess SiO_2_ of ~ 10% was also found in this specimen, which possibly derived from the incorporation of IBFC in the material. Interestingly, the addition of strain WH into OPC+IBFC+WH yielded a much higher CaCO_3_ content but lower in C_3_S than the OPC+IBFC. This suggested that an increase in CaCO_3_ was likely due to the added biogenic CaCO_3_.

**Table 3:** Percentage of mineral compositions in cementitious composites analyzed by Rietveld Refinement method.

Specimen	Percentage of mineral composition					
	Calcite (CaCO_3_)	Portlandite (Ca(OH)_2_)	Alite (Ca_3_O_5_Si)	Gypsum (CaSO_4_·2H_2_O)	Ettringite (3CaO·Al_2_O_3_·3CaSO_4_·32H_2_O)	SiO_2_
OPC	12.3 ± 0.4	23.0 ± 0.4	19.1 ± 0.6	28.5 ± 0.5	17.0 ± 0.7	ND
OPC + WH	11.1 ± 0.4	23.3 ± 0.4	17.0 ± 0.6	20.6 ± 0.6	27.9 ± 0.5	ND
OPC + IWFC	15.3 ± 0.5	28.6 ± 0.7	13.4 ± 0.8	18.3 ± 0.7	24.3 ± 0.9	ND
OPC + IWFC + WH	12.4 ± 0.5	30.8 ± 0.7	18.1 ± 0.8	18.5 ± 0.7	20.3 ± 0.9	ND
OPC + IBFC	10.3 ± 0.6	22.8 ± 0.9	20.3 ± 0.7	19.8 ± 0.5	17.3 ± 0.8	9.6 ± 0.4
OPC + IBFC + WH	17.0 ± 2.0	21.3 ± 0.7	13.9 ± 0.8	19.7 ± 0.8	20.5 ± 1.0	7.1 ± 0.6

Chemical compositions that are not detected are denoted as ND (not detected).

In the sample containing incinerated black filter cake (IBFC), the effect of biogenic CaCO_3_ on the cement hydration product was observed. Table 3 showed that the calcite content is higher than the C_3_S content, which counted for ~ 17% and ~ 14%, respectively in the OPC+IBFC+WH sample. Besides, the extra CaCO_3_ produced by bacteria may result in the formation of ettringite. Moreover, the biogenic CaCO_3_ may delay or even inhibit the carbonation process, as evidenced by a consistent amount of portlandite and an ~ 8% increase in calcite compared to the control sample without the presence of calcium carbonate from bacteria. Furthermore, X-ray diffraction (XRD) quantitative analysis revealed a 9.6% excess of silicon dioxide (SiO_2_) in the control sample (OPC+IBFC).

## Discussion

In this work, two different types of filter cakes were incinerated and then used as a cement replacement. *Lysinibacillus* sp. WH, a CaCO_3_-producing bacterium^19^, was mixed with cement as an additive aiming to compensate for a reduction of some physical properties of the cement due to IFC replacement. XRF analysis showed that the main element in IWFC was CaO (91.25%), one of the major constituents in Portland cement, allowing it a promising cement replacement. The TGA results showed that LOI of IWFC (8.56%) was much lower than that of WFC (21.22%), so the incineration condition was appropriate for preparing IWFC for use. There have been only a few studies investigating the use of IWFC as a cement replacement^11,17^ and lime-based materials^37^. Those studies indicated that the compressive strength of self-compacting concrete and cellular lightweight concrete reduced with increasing IWFC replacement ratio, while 10% by weight of IWFC replacement was the most desirable ratio due to good spreadability^17^. Moreover, the IWFC replacement ratio of less than 20% could enhance C_3_S formation, the main phase for cement strength development^16^. Our work, therefore, selected 10% IWFC as a ratio for cement replacement and then studied the effects of strain WH to improve cement strength.

Different chemical compositions were found in the case of IBFC. The main composition of IBFC was SiO_2_ (58.80%) with a minor amount of CaO (7.69%) and other elements. The summation of AlO_2_, SiO_2_ and Fe_2_O_3_ of 68.27% and the amount of SO_3_ of 0.33% in IBFC meets the requirement of the ASTM C618-15 standard characteristics of raw or calcined natural pozzolanic materials^38^. Due to its low content of CaO and the sum of AlO_2_, SiO_2_ and Fe_2_O_3_ as high as ~ 70%, IBFC has a chemical property desirably comparable to that of Class N pozzolans, which is highly reactive towards excess lime formed during the cement hydration^38^. Moreover, its LOI of as low as 0.30% also meets the ASTM C618 standard (< 12%), allowing it a good candidate for being a pozzolanic material. Therefore, IBFC has a potential for being used as a cement replacement, in which our work is the first to investigate this.

Despite different pore diameters and total pore volumes of both IFC, %void of OPC+IWFC and OPC+IBFC were not much different and only slightly higher than that of the OPC. This suggested that a replacement of cement with both types of IFC did not affect %void of the materials. Note that although the pore space of IBFC itself was very low (as can be seen from Fig. 1), %void of OPC+IBFC was higher than that of the OPC. This was likely caused by the occurrence of pore spaces between OPC and IBFC particles due to non-homogenous incorporation of the two particles within the material matrix*.* Furthermore, we found that strain WH could enhance strength of the cement replaced with IFC, while also reduce water absorption (Fig. 3) and %void (Fig. 4). Although the curing process had a significant effect on the reduction of water absorption of the OPC+IFC, the addition of strain WH (OPC+IWFC+WH and OPC+IBFC+WH) could further reduce water absorption down to the level even less than that of the OPC. This is because strain WH and its biogenic CaCO_3_ could fill up the pores, thus preventing water permeability of the materials. This effect was correlated to a decrease in %void after 28 days of curing. Our findings were in agreement with other previous works indicating that biogenic CaCO_3_ can fill and clog the pores, resulting in a decrease in the percentage of void values^25,39,40^. However, pore-filling effect due to biogenic CaCO_3_ was more pronounced when using IBFC as a cement replacement than when using IWFC. This is because CaO has both positive and negative effects on cement hydration^41^. The desirable properties of cementitious materials including high strength, low water absorption and limited pore volume are obtained not only from the reaction of CaO, but also the formation of ettringite and the nucleation reactions of the cement replacement materials^42^.

According to XRD analysis results, we found that the presence of biogenic CaCO_3_ could induce the formation of ettringite in cement samples through the hydration of tricalcium aluminate (C_3_A) which can prevent a rapid hardening of cement, as shown in the Eq. (3). Although CaCO_3_ was regarded as an inert filler to cementitious materials^43–45^, recent studies found its positive chemical effects causing the formation of additional ettringite^46,47^. CaCO_3_ can transform monosulfate of the AFm phases (Al_2_O_3_-Fe_2_O_3_-mono), a form of hydration product, into hemicarboaluminate and/or monocarboaluminate phases together with additional formation of ettringite, one of the AFt phases (Al_2_O_3_-Fe_2_O_3_-tri). This reaction then results in an increase in total volume of solid phase in the cement matrix, causing an increase of compressive strength^46–48^.

3 $$C_{3} A + gypsum \to ettringite$$

The results also showed that the calcite content in the OPC+IWFC was higher than that of the OPC+IWFC+WH even though biogenic CaCO_3_ was added in the latter material. This might be due to the additional formation of CaCO_3_ could be induced by the acceleration of the carbonation process caused by C_3_S^49^. This was likely the case in our materials as was evidenced by the presence of a lower C_3_S content in the OPC+IWFC than in the OPC+IWFC+WH. Moreover, our biogenic CaCO_3_ could accelerate the formation of C_3_S in the sample containing IWFC (OPC+IWFC+WH), in which its compressive strength was higher than that of the OPC+IWFC. This is because the bacterial cells and its biogenic CaCO_3_ can also act as nucleation surfaces for the formation of cement hydration products^50^. However, the presence of too much CaO, which is the main composition of IWFC, in the composite OPC+IWFC can cause cement expansion and disintegration, resulting in the loss of strength^51^. This is the reason why the strength of the OPC+IWFC was lower than that of the OPC. Note that a considerable improvement of compressive strength of the IWFC-cement composite could be obtained by the addition of our strain WH.

In the case of IBFC-cement composites, the strength of the OPC+IBFC was lower than that of the OPC. This might be due to an excess of SiO_2_, the main composition of IBFC, could partially substitute the matrix of cementitious material, resulting in a reduction in strength^52^. The presence of biogenic CaCO_3_ from strain WH in the OPC+IBFC+WH composite could induce the formation of ettringite and likely to inhibit the carbonation process, as evidenced by a consistent amount of portlandite and an increase in calcite content (Table 3). Furthermore, an increase in calcite content (17%) in the OPC+IBFC+WH indicated that its strength is primarily influenced by calcite (CaCO_3_), which additionally derived from the bacteria strain WH, rather than C_3_S. This implied that strain WH and its biogenic CaCO_3_ had a direct effect, both physical and chemical, in increasing strength of IFC-cementitious composites.

All of these results suggested that both IWFC and IBFC could be used as a cement replacement at least at a ratio of 10% by weight of cement. Although physical properties of the OPC+IWFC and the OPC+IBFC were impaired due to IFC replacement, the addition of strain WH and its biogenic CaCO_3_ could compensate the reduction of those properties and, in the case of the OPC+IBFC+WH, even enhance the quality of the materials to better than those of the conventional cement (OPC). Further research on the maximum ratio of IFC which can be used as a cement replacement is planned for the future. Moreover, the pozzolanic effects of IBFC when calcifying bacteria was incorporated into the cement composite is also worth investigating.

## Conclusions

This work investigated the effects of a calcifying bacterium *Lysinibacillus* sp. WH in enhancing mechanical properties of cementitious composites containing IWFC and IBFC. Key findings in our work are outlined as follows:

1. This work is the first to use IBFC, which mainly consisted of SiO_2_ (58.80%), as a cement replacement. The physicochemical properties of IBFC suggested it a Class N pozzolan according to ASTM C618 standard.
2. The use of both IWFC and IBFC as a cement replacement (10% w/w cement) caused a reduction in compressive strength, an increase in water absorption and void when compared to OPC.
3. The addition of strain WH could compensate for the loss of strength, while reducing water absorption and void of the cementitious composites, especially in the case of OPC+IBFC+WH. Note that this is the first work to use calcifying bacteria to improve mechanical properties of cementitious composites containing natural substances derived from agro-industrial wastes.
4. An increase in strength of the OPC+IBFC+WH was likely to be due to biogenic calcite rather than C_3_S, whereas C_3_S formation was induced in the presence of strain WH in the OPC+IWFC+WH.

Therefore, the finding implied that MICP technology from strain WH was effective in improving the physical properties of biocement containing agro-industrial waste. As a result, the use of incinerated sugarcane filter cake, both white and black types, from the sugar industry as a cement replacement is applicable. It provides an alternative method for reducing industrial waste and producing environmentally friendly materials while solving the challenges of future waste pollution.

## Data Availability

The data used in this work can be made available upon reasonable request to the corresponding author.
